# Breast cancer imaging in South Africa in 2018

**DOI:** 10.4102/sajr.v22i2.1666

**Published:** 2018-11-05

**Authors:** Peter K. Schoub

**Affiliations:** 1 Department of Radiology, Parklane Radiology, Johannesburg, South Africa

It is with great pride that I present the *South African Journal of Radiology* (SAJR) breast issue. Breast cancer is the most common cancer diagnosed in women in South Africa, and together with cervical cancer, constitutes the leading cause of cancer death.^1^

Early diagnosis of breast cancer is key to reduce mortality.^2^ Breast imaging is an ever-growing sub-specialty of radiology, both in South Africa and worldwide. The goal is to detect cancer changes as early as possible and to be able to accurately differentiate cancer from non-malignant breast pathology. The ability to rule out cancer, either by confirming the benignity of a lesion or by excluding the presence of any abnormality at all, is as important as showing features of malignancy.^3^

The intention with this SAJR issue is to provide a platform for local radiologists to publish breast imaging articles, and, in particular, to identify various aspects of breast radiology that are of importance in the South African context.

One of these is the high human immunodeficiency virus (HIV) prevalence in the population. So far, it has not been shown that women with HIV are at a higher risk of developing breast cancer or that they necessarily present at a later stage than non-HIV patients.^4^ There is, however, strong evidence that women with HIV develop breast cancer at a younger age. This is corroborated by Dr Minnie et al. in their retrospective study. The implication is that breast cancer screening, or at the very least, education about breast cancer, needs to be directed at younger women. HIV should possibly be considered an independent risk factor for earlier breast cancer development, and consequently, screening from a younger age.

Unfortunately, there is a desperate shortage of radiology services in the public sector in South Africa, and as a result, most women do not have access to screening mammography.^5^ At present, the guidelines from the National Department of Health regarding screening for breast cancer do not cover mammography at all, and instead, promote clinical breast examination as the primary screening test.^5,6^ Therefore, we must acknowledge that mammography screening in this country is available only to a small percentage of the population, those with private medical aids and possibly those in large city centres where tertiary-level public hospitals do offer mammograms. The topic of screening mammography remains contentious after nearly 50 years of mammography. Nonetheless, screening has been definitively shown to reduce mortality from breast cancer and the optimal starting age and intervals for mammography are becoming clearer. Dr Lipschitz has examined the literature, and composed a lucid, thoughtful breast cancer screening guideline.

One of the most frequent diagnoses for palpable breast lumps, especially in the younger age group, is that of fibroadenomas. Despite the likely benignity of breast lumps in younger women, we are seeing a growing number of high-grade cancers in young women, some of which mimic fibroadenomas.^7^ Taking into account the paucity of mammography equipment in most public health systems across the African continent, the reluctance to use mammography in younger women and the unfeasibility of performing biopsies on all masses, Dr Olarinoye-Akorede et al. have submitted a very useful study undertaken in Zaria, Nigeria, on the utility of breast ultrasound in younger patients with palpable breast lumps. In experienced hands, and abiding by certain proven descriptors, ultrasound is both highly sensitive and specific in determining malignancy in solid breast masses.^8^

Breast density as a risk factor for breast cancer is a topic that is often under-appreciated by radiologists and referring clinicians. Current data suggest that density alone is a risk factor for cancer development, while also causing a masking effect of underlying malignancy.^9^ In the United States of America (USA), it has become law in most states that women are informed of the density of their breasts on mammogram so that they can elect for additional imaging.^10^ In South Africa, most practices that offer mammography also offer breast ultrasound. Similarly, tomosynthesis mammography, which also reduces the masking effect of dense tissue, has been widely adopted. Two articles in this issue address breast density. Dr Jackie Smilg’s article reviews categorisation and implications of density on mammogram examinations. The article is an excellent summary of the supplemental imaging that should be performed in all women with dense breast tissue. Dr Minnie et al. performed a cross-sectional study comparing radiologist-determined breast density with that of automated breast density software. They were able to clearly demonstrate the unreliability of radiologist density appreciation. Taking into account the subjective appreciation of density and the implications of assigning breast density, an objective and reproducible method of density determination may well prove to be a standard of practice.^11,12^ This is an area where artificial intelligence promises to assist in disease identification.

Breast Magnetic Resonance Imaging (MRI) has become an invaluable tool in breast cancer imaging. Although the cost of the equipment and studies themselves has limited accessibility in South Africa, most privately funded patients, and a significant number of patients in public hospitals, have access to breast MRI. The national guidelines include breast MRI in screening guidelines for high-risk women.^5^ Following a review of the latest literature, I have attempted to simplify guidelines for the utilisation of breast MRI. Indications such as high-risk screening are universally accepted. Other indications such as preoperative staging, problem solving and monitoring of neoadjuvant therapy applicable in many but not all situations.^13,14^ Understanding MRI’s advantages and limitations and the situations whereby MRI will benefit patient outcomes is imperative to making correct imaging decisions.

A prospective study by Dr Cloete et al. examines the use of breast MRI in evaluating potentially benign masses in the breast. Although ultrasound remains the mainstay of investigating these lesions, the study is particularly relevant in investigating two additional factors relating to breast MRI. The first one is the application of the Kaiser score, otherwise known as the Tree flowchart. This decision-making tool, which allows a stepwise evaluation of a breast lesion based on multiple imaging features, should not replace the Breast Imaging Reporting and Data System (BIRADS) system, but rather complement it.^15,16^ It is particularly useful for radiologists who are still learning how to interpret breast MRI scans. The second topic discussed in this article is the usage of apparent diffusion coefficient (ADC) maps. There is increasing evidence that quantitative assessment of ADC values can reliably confirm benignity, and possibly malignancy in breast lesions.^17^

Dr. Pam Smilg has contributed a clinical perspective on pseudo-angiomatous stromal hyperplasia (PASH). This condition is seen not infrequently, and can mimic carcinoma both clinically and radiologically.^18^ This article shows us examples and reminds all breast imagers to consider PASH in a differential diagnosis and realise that the diagnosis of PASH may be concordant with relatively suspicious imaging findings.

Finally, we have a fascinating article from Ramaema and Hift that looks at the utility of multiparametric breast MRI (Contrast enhancement, T2 weighted and DWI/ADC sequences) in differentiating breast cancer from tuberculosis of the breast. There is growing emphasis on qualitative and quantitative multiparametric imaging in breast MRI^19^. The qualities of various different sequences can be combined to provide highly specific diagnostic information.

I hope you will find this issue of interest. I also hope that it will inspire some of you who have an interest in breast imaging to submit your own breast-related content for publishing. Up until now, we have based most of our guidelines and protocols on data from the USA and Europe. It will be far more beneficial to have data from within Southern Africa upon which to base our breast imaging decisions, improve our diagnostic skills and help optimise breast cancer outcomes throughout our region.
